# Cardiovascular Magnetic Resonance in Peripartum Cardiomyopathy: Comparison with Idiopathic Dilated Cardiomyopathy

**DOI:** 10.3390/diagnostics11101752

**Published:** 2021-09-24

**Authors:** Joanna Petryka-Mazurkiewicz, Karolina Kryczka, Łukasz Mazurkiewicz, Barbara Miłosz-Wieczorek, Mateusz Śpiewak, Magdalena Marczak, Jan Henzel, Jacek Grzybowski, Marcin Demkow, Zofia Dzielińska

**Affiliations:** 1 Department of Coronary and Structural Heart Diseases, National Institute of Cardiology, 04-628 Warsaw, Poland; kkryczka@ikard.pl (K.K.); jhenzel@ikard.pl (J.H.); mdemkow@ikard.pl (M.D.); zdzielinska@ikard.pl (Z.D.); 2 Magnetic Resonance Unit, National Institute of Cardiology, 04-628 Warsaw, Poland; bmilosz@ikard.pl (B.M.-W.); mspiewak@ikard.pl (M.Ś.); mmarczak@ikard.pl (M.M.); 3 Department of Cardiomyopathy, National Institute of Cardiology, 04-628 Warsaw, Poland; lmazurkiewicz@ikard.pl (Ł.M.); jgrzybowski@ikard.pl (J.G.)

**Keywords:** peripartum cardiomyopathy, dilated cardiomyopathy, cardiovascular magnetic resonance, feature tracking, late gadolinium enhancement

## Abstract

Background: Peripartum (PPCM) and dilated (DCM) cardiomyopathies are distinct forms of cardiac disease that share certain aspects in clinical presentation. Aim: We hypothesized that different cardiac structural changes underlie PPCM and DCM, and we aimed to investigate them with cardiovascular magnetic resonance (CMR). Methods: We included 21 PPCM patients (30.5 ± 5.9 years) and 30 female DCM patients (41.5 ± 16.8 years) matched for left ventricular ejection fraction. Biventricular and biatrial volumetric and functional parameters were assessed along with ventricular and atrial strain indices based on feature-tracking techniques. The presence of late gadolinium enhancement (LGE) was also assessed. Results: In PPCM, the left ventricular (LV) stroke volume index was lower (*p* = 0.04), right atrial (RA) minimal and pre-systolic volumes were higher (*p* < 0.01 and *p* = 0.02, respectively), and the total RA ejection fraction was lower (*p* = 0.02) in comparison to DCM. Moreover, in PPCM, the LV global longitudinal strain (*p* = 0.03), global circumferential strain rate (*p* = 0.04), and global longitudinal strain rate (*p* < 0.01) were less impaired than in DCM. Both PPCM and DCM patients with LGE had more dilated ventricles and more impaired LV and left atrial function than in PPCM and DCM patients without LGE. Conclusions: Subtle differences appear on CMR between PPCM and DCM. Most importantly, the RA is larger and more impaired, and LV global longitudinal strain is less reduced in PPCM than in DCM. Furthermore, similarly to DCM, PPCM patients with LGE have more dilated and impaired ventricles than patients without LGE.

## 1. Introduction

Peripartum cardiomyopathy (PPCM) and dilated cardiomyopathy (DCM) are two cardiac conditions that share some aspects in clinical presentation, such as dilated ventricles, reduced systolic function, and arrhythmias [1]. However, PPCM is a rare cardiac condition that presents itself during late pregnancy or during the early postpartum period, with a clinical course that is distinct from that of idiopathic DCM in young women [2]. Patients with PPCM typically present in their early 30s, while patients with DCM usually present later in life, unless genetically predisposed. While heart muscle function in DCM never fully recovers but slowly deteriorates further, the clinical course of PPCM may vary between rapid cardiac deterioration or complete recovery. Between 20% and 60% of patients with PPCM recover LV function [3,4]. However, the mortality rate of PPCM remains high—from 11% to 32% [3,4]. Patients with DCM have a decreasing survival rate, with 90% of patients surviving at 1 year, 50% at 5 years, and 33% at 10 years [5]. Already in the 1960s of the twentieth century, it was observed that some cases of PPCM cluster in families [6]. A genetic cause for DCM and PPCM has been demonstrated before [7]. It has been reported that approximately 15% to 20% of PPCM patients carry cardiomyopathy-causing mutations, mainly in the *TTN*, *MYH7*, and *SCN5A* genes [8]. Mutations in these genes are associated with DCM, as well [8].

Cardiac magnetic resonance (CMR) imaging is a recognized radiation-free technique used to assess cardiac structure and function [9]. Cine CMR is a highly accurate and reproducible technique for the determination of cardiac volumes, function, and mass [10]. Furthermore, CMR feature tracking (CMR-FT) provides detailed analysis of biventricular and biatrial mechanics. Late gadolinium enhancement (LGE) CMR is considered the gold standard for assessing focal myocardial fibrosis. Although CMR imaging is used for the diagnosis and detection of myocardial damage in various heart diseases, there are limited data on its diagnostic or prognostic usefulness in PPCM [11].

On the basis of differences in disease onset and progression, we hypothesized that different cardiac structural changes underlie PPCM and DCM, and we aimed to investigate them with CMR.

## 2. Materials and Methods

This study was approved by the Institutional Ethics Committee, and written informed consent to participate in the study was obtained from all subjects.

### 2.1. Study Population

The study cohort consisted of subjects with PPCM and female subjects with DCM matched for left ventricular ejection fraction, who underwent clinically indicated CMR in our center between 2014 and 2020. PPCM was defined according to the ESC criteria as cardiomyopathy presenting towards the end of pregnancy or in the months following delivery where no other cause of heart failure was found [12]. DCM was diagnosed based on the revised ESC definition that includes the presence of left ventricular (LV) or biventricular dilatation and systolic dysfunction in the absence of abnormal loading conditions (hypertension and valve disease) or the presence of coronary artery disease sufficient to cause global systolic impairment [1].

### 2.2. CMR Examination

**Figure 1:**
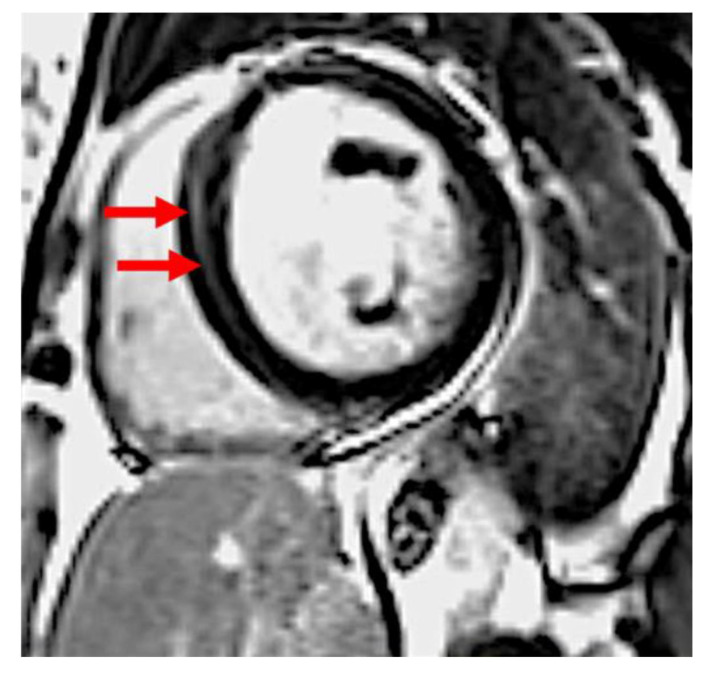
Late gadolinium enhancement in basal antero-septal wall in a patient with peripartum cardiomyopathy. Arrows indicate abnormal signal in the septum.

Cardiovascular magnetic resonance imaging was performed by using a 1.5-T scanner (Sonata and Avanto fit, Siemens, Erlangen, Germany). Cine images were acquired by breath-hold, electrocardiographic-gated, segmented k-space steady-state free-precession (SSFP) technique using 25 phases per cardiac cycle. LGE images were obtained in the long-axis and short-axis imaging planes by using a breath-hold segmented inversion recovery sequence implemented 10–15 min after intravenous administration of 0.1 mmol/kg of gadobutrol (Gadovist, Bayer, Berlin, Germany). The presence of left ventricular LGE was initially visually assessed by two independent experienced observers (JPM and LM). If positive, quantification was performed using the using CVi42 software (Circle Cardiovascular Imaging Inc., Calgary, AB, Canada) with a signal intensity threshold of >6 standard deviations above the remote myocardium (Figure 1) [13]. The extent of LGE was presented as a percentage of the total LV mass.

### 2.3. Image Analysis

SSFP images were used to calculate left (LV) and right ventricular (RV) volumes and ejection fractions (EF) with the use of dedicated software (Syngo via. Siemens, Erlangen, Germany), as previously described [14]. Left atrial (LA) and right atrial (RA) volumetric and functional calculations were performed separately by two independent and skilled observers (JPM and LM) according to a previously described protocol [15].

### 2.4. Feature Tracking

CMR-FT analysis was carried out by using CVi42 (Circle Cardiovascular Imaging Inc, Calgary, AB, Canada). LV and RV end-diastolic endocardial and LV epicardial contours were drawn automatically. RV epicardial contours were manually traced at the end-diastole. All contours were propagated, with manual readjustments performed as required. The global longitudinal strain and global longitudinal strain rate parameters were obtained from the 4-chamber view for the RV (RVGLS and RVGLS rate, respectively) and from the 2-chamber and 4-chamber views for the LV (LVGLS and LVGLS rate, respectively). Circumferential and radial strains and strain rates parameters for both the RV (RVCR, RVCR rate, RVR, and RVR rate) and the LV (LVCR, LVCR rate, LVR, and LVR rate) were determined in the short-axis view in the basal and mid-sections of the ventricles. Due to the known tendency for artifacts and lower muscle thickness in periapical slices, these images were not included in the FT analysis. The basal slice in the short-axis view was defined as the first slice below the atrioventricular level showing a circumferential myocardial enclosing. The midventricular slice was localized at the level of both papillary muscles. The values of the circumferential and radial strains and strain rates obtained in each slice of RV and LV were averaged.

**Figure 2:**
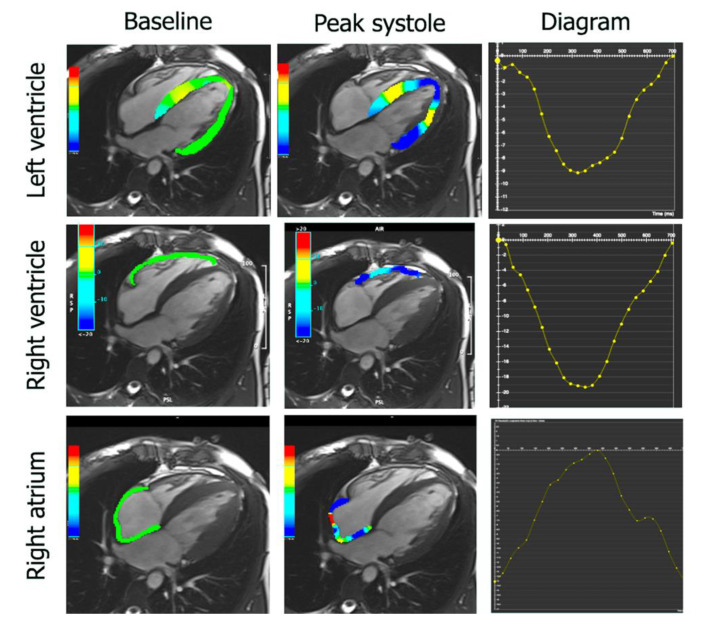
Longitudinal strain analysis in left and right ventricles and in the right atrium. Cardiovascular magnetic resonance feature tracking with the use of CVi42 in a patient with peripartum cardiomyopathy.

The atrial strain and strain rates were also analyzed (Figure 2). LA endocardial borders in the two-chamber and four-chamber views, and RA borders in the four-chamber view were automatically drawn and propagated, with manual readjustments performed as required. Three aspects of LA and RA mechanics were analyzed: passive global longitudinal strain (GLS passive), active global longitudinal strain (GLS active), and total global longitudinal strain, which is the sum of the passive and active strains (GLS total). Accordingly, three strain rate parameters were evaluated: the peak positive strain rate (SRS), the peak early negative strain rate (SRE), and the peak late negative strain rate (SRA).

### 2.5. Statistics

All continuous variables were expressed as the mean ± standard deviation (SD) or as the median and interquartile range and were tested for normal distribution with the use of the Kolmogorov–Smirnov test. Comparisons between groups were performed using the Student’s *t*-test or the Wilcoxon–Mann–Whitney U test for continuous variables, and chi-square or Fisher’s exact tests were used for categorical variables where appropriate. Intraobserver and interobserver variability were evaluated by using the Bland–Altman test and expressed as the mean difference ± SD, intraclass correlation coefficients (ICCs) with a 95% confidence interval (CI), and the coefficient of variation (CoV). A two-sided *p* value of less than 0.05 was considered to indicate statistical significance. Statistical analyses were performed with MedCalc 12.1.4.0 software (MedCalc, Mariakerke, Belgium).

## 3. Results

Overall, we included 21 patients with PPCM and 30 female patients with a diagnosis of DCM, matched for ejection fraction (35.9 ± 13.7% vs. 35.2 ± 11.2%, *p* = ns). Expectedly, DCM patients were older than PPCM patients (41.5 ± 16.8 vs. 30.5 ± 5.9 years, *p* = 0.03). PPCM patients more often presented with severe symptoms of heart failure as assessed by NYHA classification (2.97 vs. 2.20, *p* = 0.001) and had higher resting heart rates (83.1 ± 15.9 vs. 69.5 ± 11.4 beats/min, *p* = 0.002) as they less frequently received β-blocker therapy in comparison to DCM patients (81% vs. 100%, *p* = 0.02)

During follow up, among the 21 PPCM patients, one patient underwent heart transplantation, one patient required temporary left ventricular assist device implantation, and one patient underwent biventricular assist device implantation with subsequent explantation following clinical improvement. Apart from the three patients mentioned above, the left ventricular ejection fraction remained impaired (below 50%) in another four patients with PPCM.

There were no differences in the left and right ventricular volumetric and functional parameters, apart from LV stroke volume index, which was higher in DCM than compared to PPCM (50.3 ± 11.8 mL/m^2^ vs. 44.5 ± 10.5 mL/m^2^, *p* = 0.04). Similarly, there were no differences in left atrial volumetric and functional parameters. However, in patients with PPCM, right atrial minimal and pre-systolic volumes were higher than in women with DCM (47.2 ± 16.0 mL vs. 37.2 ± 8.2 mL, *p* < 0.01 and 68.7 ± 18.3 mL vs. 58.4 ± 13.9 mL, *p* = 0.02, respectively). Moreover, the total RA EF was lower in PPCM in comparison to DCM (42.4 ± 10.0 mL vs. 48.6 ± 9.8 mL, *p* = 0.02). There were no differences with regards to the frequency and the extent of LGE between PPCM patients and women with DCM (Table 1).

The distribution of LGE was similar in both groups. In the PPCM group, most of patients (n = 8, 89%) presented with mid-wall fibrosis of the interventricular septum and/or the inferior insertion point. Only one PPCM patient was found to have subendocardial LGE on CMR. Similarly, mid-wall fibrosis was detected in 10 (55%) female DCM patients. In the remaining eight (45%) DCM patients, LGE of both mid-wall and subendocardial distribution was observed.

Strain analysis showed that patients with PPCM had less impaired left ventricular GLS (−9.6 ± 3.9 vs. −7.7 ± 2.6, *p* = 0.03) and GLS rates (−0.55 ± 0.14 vs. −0.45 ± 0.12, *p* <0.01) in comparison with female patients with DCM. Furthermore, PPCM patients had less impaired left ventricular GCS rates (−0.64 ± 0.26 vs. −0.53 ± 0.13, *p* = 0.04). However, there were no differences in the right ventricular and biatrial mechanical indices between the groups (Table 2).

Expectedly, both PPCM and DCM patients with LGE had more dilated left ventricles and more impaired LV function in comparison with PPCM and DCM patients without LGE (Table 3). Additionally, female DCM patients with fibrosis detected by CMR had higher LV mass (*p* = 0.04) and LV mass index (*p* < 0.01) in comparison to DCM patients without fibrosis.

Regarding the RV, PPCM and DCM patients with LGE had higher RV end-systolic volume (PPCM: *p* = 0.01; DCM: *p* = 0.01), RV end-systolic volume index (PPCM: *p* = 0.01; DCM: *p* < 0.01), and more impaired RV EF (PPCM: *p* = 0.01; DCM: *p* = 0.02). Additionally, female DCM patients with LGE also had higher RV end-diastolic volume (*p* = 0.04) and RV end-diastolic volume index (*p* = 0.02) when compared to DCM women without LGE.

Furthermore, PPCM patients with LGE on CMR had larger LA (area: *p* < 0.01; LAV max: *p* = 0.01; LAV min: *p* < 0.01; LAV pre-ac: *p* < 0.01) and more impaired total (*p* < 0.01) and passive (*p* < 0.01) LA EF than PPCM patients without LGE. Female patients with DCM presenting with LGE had lower total LA EF (*p* = 0.02) than compared to women without LGE. While there were no differences in RA volumetric and functional indices in the PPCM group, DCM patients with LGE had smaller right atria (*p* < 0.01) and lower total (*p* = 0.01) and passive (*p* = 0.01) RA EF in comparison with DCM patients without LGE. 

The analysis of all left ventricular strain and strain rate parameters showed that they were more impaired in patients with LGE than in patients without LGE in both PPCM (GLS: *p* < 0.01; GCS: *p* = 0.01; GRS: *p* < 0.01; GCS rate: *p* = 0.01; GRS rate: *p* = 0.01) and DCM groups (GLS: *p* = 0.01; GCS: *p* < 0.01; GRS: *p* < 0.01; GCS rate: *p* < 0.01; GRS rate: *p* = 0.04). In PPCM group the mean value of LV GLS rate was higher in patients without LGE than in LGE positive patients but the difference did not meet statistical significance (p = 0.053). 

With regards to RV mechanics, PPCM and DCM patients with LGE had more impaired RV GCS (PPCM: *p* = 0.01; DCM: *p* = 0.01), RV GRS (PPCM: *p* < 0.01; DCM: *p* = 0.03), and GRS rate (PPCM: *p* = 0.04; DCM: *p* = 0.01) in comparison to PPCM and DCM patients without LGE. Additionally, female DCM patients with LGE had more impaired GCS rate in comparison with DCM patients without LGE (−0.39 ± 0.27 vs. −0.62 ± 0.19, *p* = 0.01).

Similarly, some of the LA mechanical indices were better preserved in PPCM (LA GLS total: *p* < 0.01; LA GLS active: *p* < 0.01; LA SRS: *p* = 0.01) and DCM (LA GLS total: *p* < 0.01; LA GLS active: *p* < 0.01) patients without LGE than in PPCM and DCM patients with LGE. There were no differences in the right atrial mechanical indices in PPCM patients with and without LGE. While in DCM patients with fibrosis, the RA GLS total (*p* = 0.01), RA GLS passive (*p* < 0.01), and RA SRS (*p* = 0.02) were more impaired than in the group of DCM patients without LGE (Table 4).

The indices of reproducibility for atrial and ventricular strain and strain rate parameters were satisfactory—intraobserver and interobserver ICCs ranged between 0.50 and 0.99 for all components of myocardial performance (Supplementary Materials, Table S1).

## 4. Discussion

Our main findings can be summarized as follows:

1. Patients with PPCM have lower LV stroke volume index and higher RA volume with lower RA total ejection fraction in comparison to women with DCM.
2. In patients with PPCM, some indices of LV mechanics such as LV GLS and LV GLS rate, as well as LV GRS rate, are less impaired than in DCM women.
3. Both PPCM and DCM female patients with LGE have more dilated and impaired left and right ventricles in comparison to patients without LGE on CMR imaging.
4. Almost all LV and RV mechanical indices are more impaired in patients with LGE in comparison to patients without LGE in both PPCM and DCM groups.

### 4.1. CMR for Diagnosis and Prognostication

The role of CMR in the diagnosis and follow up process in patients with DCM and PPCM is well established. Firstly, an accurate assessment of RV size and function can be challenging by using other techniques, such as echocardiography, because of its complex and variable shape. Reduced RV ejection fraction on CMR is an independent predictor of all-cause mortality and adverse heart failure outcomes in DCM and PPCM [12,16]. In detail, an impaired RV is associated with a dilation of both ventricles and lower LVEF, suggesting more extensive biventricular cardiac involvement in PPCM [17]. CMR provides the gold standard noninvasive assessment of both RV and LV size and function because of its three-dimensional capabilities [18]. In our study, PPCM patients had more reduced LVSVI than DCM patients. This finding may be explained by the fact that myofilaments from PPCM patients show more impaired function than myofilaments from patients with DCM [1]. The reason for that may be the lack of expression of fetal EH-myomesin in PPCM. EH-myomesin is responsible for stabilizing heart muscle function in DCM, but the presence of this remodeling disables DCM patients from total recovery that is observed in PPCM [1].

CMR also allows accurate quantification of left and right atrial volumes by using the biplane area-length method [19] and is superior than other noninvasive imaging methods because of its excellent endocardial border definition and multiplanar imaging ability, even in the presence of atrial fibrillation [20]. It has been proposed that the degree of LA enlargement acts as a barometer of diastolic dysfunction; therefore, the LA size is used to predict heart failure outcomes [21]. It has been previously demonstrated that indexed LA volume calculated by using CMR independently predicts cardiac transplant-free survival in DCM [22]. Interestingly, in our study, PPCM patients had larger right atrium, but not LA, with more impaired function in comparison to DCM patients. This finding may be associated with increased blood volume, as a physiological adaptation during pregnancy and peripartum period affects RA more than the LA volume. Furthermore, the impairment of RA function may reflect more severe diastolic dysfunction of overloaded RV. The LA was enlarged to a comparable degree in both PPCM and DCM patients.

Finally, given its accuracy in the assessment of cardiac muscle function, CMR is the method of choice for the follow up of patients with DCM after pharmacologic and surgical interventions [23] and should be applied to PPCM patients as well [9]. Given the favorable interobserver variability compared with other methods of assessment, the use of CMR can reduce the sample size required, reducing the overall cost and time needed to complete a research study [24]. In our study, the reproducibility of strain and strain-rate parameters was satisfactory, especially with regards to volumetric measurements.

### 4.2. LV Fibrosis in PPCM

Data on the role of LGE in PPCM are limited [25]. Importantly, fibrosis was detected histologically in samples obtained during heart transplantation from patients with PPCM [1]. In our study, LGE was noted in 43% patients with PPCM, which is similar to data reported by Arora et al., who demonstrated the presence of LGE in 40% of PPCM patients on CMR [26]. It has been suggested that LGE in PPCM is time dependent, with LGE observed early in the course of the disease before LV function recovery [27]. Furthermore, other authors have reported the link between LGE in PPCM and an unfavorable prognosis with slower recovery, higher risk of prolonged or permanent systolic dysfunction, and higher rate of developing heart failure exacerbation in future pregnancies [28]. Likewise, the presence of fibrosis in DCM is known to be associated with adverse ventricular remodeling and increased all-cause mortality [29]. Therefore, the need for CMR with the assessment of fibrosis in the early course of PPCM, if possible, should be emphasized [9]. In PPCM, the degree of fibrosis may help advise on future pregnancies after recovery. Expectedly, in our study, PPCM patients with LGE, analogously to female DCM patients with LGE, presented with increased dilation of both ventricles along with impaired biventricular function in comparison to patients without LGE. A large prospective study of the prognostic value of fibrosis on CMR in PPCM is needed [30].

### 4.3. LV Fibrosis and Myocardial Mechanics

CMR-derived feature tracking strain analysis represents another promising tool for improving PPCM patients’ risk stratification. There is evidence which suggests that FT parameters can predict survival in DCM and improve risk stratification beyond clinical parameters, biomarkers, LVEF, and LGE [31]. Similarly, in patients with PPCM, a study demonstrated that echocardiographic measures of GLS and GCS were additive to routine LV functional measures and clinical factors, such as black race, for identifying PPCM patients at risk [32]. Specifically, patients with more impaired LV global strain at presentation were at increased risk for impaired LV recovery, death, or LVAD implantation. Other results demonstrated that LV longitudinal deformation is globally impaired in patients with PPCM, regardless of the LVEF values [33]. Our study showed that almost all LV and RV mechanical indices were more impaired in patients with fibrosis compared to patients without fibrosis in both the PPCM and DCM groups. The difference between PPCM patients with and without LGE with regards to LV GLS rate was at the limit of significance probably due to small number of patients studied and should be evaluated in further research.

In PPCM patients, LV GLS and LV GLS rates, as well as the LV GRS rate, were found to be less impaired than in women with DCM. This fact may be associated with shorter disease duration in the PPCM group despite the matched ejection fraction.

While there are data that echo-derived LVGLS was the most accurate method for detecting LV fibrosis as assessed by histopathologic examination in patients undergoing heart transplantation [34], this is the first study to link LGE with the severity of RV and LV deformation abnormalities in PPCM.

### 4.4. Study Limitations

The limitations of our study are related to its design. It is a single center retrospective study with a relatively small number of patients with PPCM. It must also be noted that CMR mapping techniques were not applied to our cohort, whereas they are now considered important in the diagnosis of PPCM. Our results may suggest the use of biventricular strain parameters for predicting fibrosis severity in PPCM [14]. However, due to the small sample size, we did not assess any association between strain values and outcomes.

## 5. Conclusions

In conclusion, some subtle differences in CMR imaging are present between PPCM patients and women with DCM. Most importantly, the right atrium is larger and more impaired in PPCM in comparison to DCM, and the left ventricular global longitudinal strain is less reduced in PPCM than in DCM. Furthermore, similarly to DCM, PPCM patients with LGE on CMR have more dilated and impaired left and right ventricles than compared to patients without LGE. The results of this study provide new insights into the cardiac phenotype of PPCM in comparison to DCM and may thereby provide valuable information to complement the clinical findings needed in establishing a diagnosis of PPCM. This study also shows that CMR can be a useful and robust tool to help differentiate between PPCM and DCM if the clinical picture is ambiguous.

## Figures and Tables

**Table 1:** Volumetric and functional CMR characteristics between patients with PPCM and female patients with DCM.

	PPCM n = 21	Female DCM n = 30	*p*
Age (years)	30.5 ± 5.9	41.5 ± 16.8	0.03
BSA (m^2^)	1.76 ± 0.19	1.77 ± 0.16	0.91
Diabetes n (%)	0 (0%)	2 (7%)	0.23
Arterial hypertension (%)	3 (14%)	2 (7%)	0.17
NYHA class	2.97	2.20	0.001
HR (beats/min)	83.1 ± 15.9	69.5 ± 11.4	0.002
SBP (mmHg)	107.3 ± 19.2	110.9 ± 14.1	0.47
DBP (mmHg)	69.2 ± 15.0	68.9 ± 8.9	0.93
Troponin T (ng/mL)	10.7 (0.001–6800)	5.45 (0.001–23.7)	0.34
NT-proBNP (pg/mL)	3007 (27–35,000)	2167 (79–3036)	0.048
B-blocker n (%)	17 (81%)	30 (100%)	0.02
ACE-I/ARB n (%)	20 (95%)	30 (100%)	0.16
Aldosterone antagonist n (%)	15 (71%)	20 (66%)	0.39
Left ventricle			
LVEDV (mL)	245.5 ± 97.4	266.9 ± 61.6	0.36
LVESV (mL)	167.1 ± 89.7	178.2 ± 68.6	0.63
LVSV (mL)	78.6 ± 22.0	88.5 ± 20.3	0.12
LVEF (%)	35.9 ± 13.7	35.2 ± 11.2	0.85
LVM (g)	125.9 ± 33.2	126.1 ± 36.2	0.99
LVEDVI (mL/m^2^)	138.3 ± 50.1	151.7 ± 35.5	0.29
LVESVI (mL/m^2^)	93.7 ± 48.6	101.3 ± 39.1	0.56
LVSVI (mL/m^2^)	44.5 ± 10.5	50.3 ± 11.8	0.04
LVMI (g/m^2^)	72.2 ± 16.8	71.2 ± 19.7	0.86
Right ventricle			
RVEDV (mL)	162.9 ± 44.7	152.1 ± 39.8	0.39
RVESV (mL)	96.2 ± 43.8	93.2 ± 45.1	0.82
RVSV (mL)	66.8 ± 23.4	59.1 ± 20.2	0.24
RVEF (%)	42.6 ± 13.2	41.1 ± 14.3	0.74
RVEDVI (mL/m^2^)	92.7 ± 23.4	85.9 ± 23.0	0.33
RVESVI (mL/m^2^)	54.8 ± 25.3	52.0 ± 23.4	0.70
RVSVI (mL/m^2^)	37.6 ± 11.5	34.1 ± 13.6	0.37
Left atrium			
LA area (cm^2^)	26.1 ± 7.5	27.2 ± 5.4	0.57
LAV max (mL)	93.1 ± 34.7	93.9 ± 21.2	0.93
LAV min (mL)	50.2 ± 28.5	46.2 ± 19.8	0.61
LAV pac (mL)	74.6 ± 35.8	73.5 ± 24.2	0.91
Total LAEF (%)	48.9 ± 13.6	52.3 ± 12.1	0.41
Passive LAEF (%)	23.0 ± 12.0	23.0 ± 12.1	0.99
Active LAEF (%)	33.9 ± 12.6	37.8 ± 14.6	0.40
Right atrium			
RA area	22.3 ± 4.2	19.8 ± 3.6	0.01
RAV max (mL)	82.0 ± 22.0	74.3 ± 24.2	0.26
RAV min (mL)	47.2 ± 16.0	37.2 ± 8.2	<0.01
RAV pac (mL)	68.7 ± 18.3	58.4 ± 13.9	0.02
Total RAEF (%)	42.4 ± 10.0	48.6 ± 9.8	0.02
Passive RAEF (%)	15.6 ± 8.7	20.0 ± 12.5	0.24
Active RAEF (%)	31.7 ± 10.3	35.6 ± 8.0	0.19
LGE			
LGE n (%)	9 (43%)	18 (60%)	0.22
LGE % (IQR)	1.7% (0–2.9%)	2.3% (0–4.5%)	0.63

ACE-I—angiotensin converting enzyme inhibitor; ARB—angiotensin receptor blocker; BSA—body surface area; CMR—cardiovascular magnetic resonance; DBP—diastolic blood pressure; HR—heart rate; LA—left atrium; LAEF—left atrial ejection fraction; LAV max—left atrial maximal volume; LAV min—left atrial minimal volume; LAV pac—left atrial pre-systolic volume; LVEDV—left ventricular end diastolic volume; LVEDVI—left ventricular end diastolic volume index; LVEF—left ventricular ejection fraction; LVESV—left ventricular end systolic volume; LVESVI—left ventricular end systolic volume index; LGE—late gadolinium enhancement; LVM—left ventricular myocardial mass; LVSV—left ventricular stroke volume; LVMI—left ventricular myocardial mass; LVSVI—left ventricular stroke volume index; NT-proBNP—N-terminal pro b-type natriuretic peptide; NYHA—New York Heart Association heart failure class; RA—right atrium; RAEF—right atrial ejection fraction; RAV max—right atrial maximal volume; RAV min—right atrial minimal volume; RAV pac—right atrial pre-systolic volume; RVEDV—right ventricular end diastolic volume; RVEDVI—right ventricular end diastolic volume index; RVESV—right ventricular end systolic volume; RVESVI—right ventricular end systolic volume index; RVEF—right ventricular ejection fraction; RVSV—right ventricular stroke volume; RVSVI—right ventricular stroke volume index; SBP—systolic blood pressure.

**Table 2:** CMR-FT derived strain parameters in patients with PPCM and female patients with DCM.

	PPCM n = 21	Female DCM n = 30	*p*
Left ventricle			
LV GRS (%)	14.1 ± 6.4	13.9 ± 4.9	0.90
LV GCS (%)	−11.0 ± 4.6	−9.8 ± 3.3	0.32
LV GLS (%)	−9.6 ± 3.9	−7.7 ± 2.6	0.03
LV GRS rate (s^−1^)	0.91 ± 0.44	0.76 ± 0.21	0.22
LV GCS rate (s^−1^)	−0.64 ± 0.26	−0.53 ± 0.13	0.04
LV GLS rate (s^−1^)	−0.55 ± 0.14	−0.45 ± 0.12	<0.01
Right ventricle			
RV GRS (%)	14.1 ± 6.2	13.2 ± 8.9	0.70
RV GCS (%)	−9.1 ± 3.8	−8.3 ± 6.4	0.62
RV GLS (%)	−14.7 ± 7.1	13.8 ± 6.8	0.68
RV GRS rate (s^−1^)	1.01 ± 0.49	0.83 ± 0.47	0.21
RV GCS rate (s^−1^)	−0.54 ± 0.28	−0.48 ± 0.26	0.49
RV GLS rate (s^−1^)	−1.01 ± 0.40	−0.86 ± 0.31	0.20
Left atrium			
LA GLS total (%)	17.5 ± 9.2	19.8 ± 9.1	0.43
LA GLS passive (%)	9.3 ± 5.1	10.4 ± 4.1	0.46
LA GLS active (%)	8.2 ± 6.3	8.7 ± 4.4	0.74
LA SRS (s^−1^)	0.72 ± 0.35	0.75 ± 0.40	0.80
LA SRE (s^−1^)	−0.66 ± 0.41	−0.83 ± 0.50	0.25
LA SRA (s^−1^)	−0.70 ± 0.40	−0.62 ± 0.36	0.57
Right atrium			
RA GLS total (%)	26.6 ± 9.6	26.5 ± 9.1	0.96
RA GLS passive (%)	11.2 ± 6.3	11.4 ± 5.8	0.94
RA GLS active (%)	15.4 ± 8.1	13.2 ± 5.8	0.32
RA SRS (s^−1^)	1.18 ± 0.40	1.14 ± 0.42	0.80
RA SRE (s^−1^)	−0.59 ± 0.32	−0.72 ± 0.48	0.34
RA SRA (s^−1^)	−1.00 ± 0.67	−0.89 ± 0.39	0.58

GCS—global circumferential strain; GLS—global longitudinal strain; GRS—global radial strain, LA—left atrium; LV—left ventricle; RA—right atrium; RV—right ventricle; SRA—late negative strain rate; SRE—early negative strain rate; SRS—peak positive strain rate.

**Table 3:** Volumetric and functional CMR characteristics between female patients with PPCM and DCM with and without LGE.

	PPCM without LGE n = 12 (67%)	PPCM with LGE n = 9 (43%)	*p*	DCM without LGE n = 12 (40%)	DCM with LGE n = 18 (60%)	*p*
Age (years)	29.9 ± 6.3	31.4 ± 5.7	ns	44.4 ± 14.8	39.6 ± 18.6	0.44
Left ventricle						
LVEDV (mL)	201.9 ± 81.1	307.7 ± 88.1	0.022	236.0 ± 63.3	287.4 ± 54.3	0.02
LVESV (mL)	122.6 ± 70.0	230.6 ± 78.2	0.009	141.5 ± 56.9	202.7 ± 67.2	0.01
LVSV (mL)	79.5 ± 25.6	77.3 ± 17.5	0.84	94.3 ± 15.8	84.7 ± 22.9	0.20
LVEF (%)	42.5 ± 13.0	26.4 ± 8.1	0.007	41.2 ± 8.3	31.2 ± 11.4	<0.01
LVM (g)	121.3 ± 38.3	132.4 ± 25.6	0.51	110.7 ± 31.9	136.3 ± 36.9	0.04
LVEDVI (mL/m^2^)	114.7 ± 39.8	172.0 ± 45.5	0.015	129.5 ± 30.5	166.6 ± 31.8	<0.01
LVESVI (mL/m^2^)	69.2 ± 37.1	128.7 ± 42.3	0.008	77.3 ± 28.1	117.3 ± 38.1	<0.01
LVSVI (mL/m^2^)	45.3 ± 11.9	43.3 ± 8.8	0.71	52.2 ± 9.1	49.1 ± 13.6	0.49
LVMI (g/m^2^)	69.2 ± 17.6	76.4 ± 15.7	0.40	60.3 ± 13.8	78.4 ± 20.2	<0.01
Right ventricle						
RVEDV (mL)	146.9 ± 45.1	185.7 ± 35.2	0.08	134.8 ± 41.7	163.6 ± 36.2	0.04
RVESV (mL)	75.0 ± 27.9	126.4 ± 46.2	0.012	70.3 ± 35.1	108.4 ± 46.2	0.01
RVSV (mL)	72.0 ± 25.5	59.4 ± 19.5	0.29	64.5 ± 21.3	55.4 ± 20.3	0.23
RVEF (%)	49.1 ± 8.8	33.4 ± 13.5	0.011	49.2 ± 13.7	35.8 ± 16.3	0.02
RVEDVI (mL/m^2^)	83.8 ± 22.9	105.4 ± 18.7	0.057	74.0 ± 23.5	93.9 ± 20.1	0.02
RVESVI (mL/m^2^)	42.7 ± 16.6	72.0 ± 26.5	0.013	38.3 ± 18.0	61.1 ± 22.9	<0.01
RVSVI (mL/m^2^)	40.4 ± 12.3	33.6 ± 5.4	0.24	35.8 ± 14.1	32.9 ± 14.1	0.56
Left atrium						
LA area (cm^2^)	21.4 ± 4.7	32.7 ± 5.4	0.0003	26.5 ± 5.3	27.7 ± 5.8	0.57
LAV max (mL)	73.5 ± 28.2	115.6 ± 29.8	0.015	90.6 ± 17.8	96.1 ± 24.6	0.53
LAV min (mL)	30.7 ± 14.5	72.4 ± 25.1	0.001	37.8 ± 16.7	51.5 ± 21.4	0.09
LAV pac (mL)	51.5 ± 23.7	101.1 ± 30.2	0.004	68.0 ± 24.4	77.0 ± 25.9	0.37
Total LAEF (%)	58.3 ± 10.1	38.1 ± 8.6	0.001	59.3 ± 11.7	47.9 ± 11.3	0.02
Passive LAEF (%)	31.3 ± 8.6	13.5 ± 8.2	0.001	26.6 ± 14.4	20.6 ± 12.3	0.25
Active LAEF (%)	39.0 ± 14.2	28.2 ± 8.8	0.11	44.4 ± 12.8	33.7 ± 15.5	0.07
Right atrium						
RA area	21.7 ± 4.9	23.3 ± 3.1	0.46	22.2 ± 3.1	18.0 ± 2.9	<0.01
RAV max (mL)	84.0 ± 26.3	79.7 ± 18.8	0.73	82.1 ± 16.9	69.4 ± 21.6	0.11
RAV min (mL)	48.0 ± 16.9	46.3 ± 16.8	0.84	40.0 ± 5.9	35.5 ± 9.6	0.18
RAV pac (mL)	67.1 ± 19.1	70.6 ± 19.6	0.73	61.0 ± 14.9	56.7 ± 14.5	0.49
Total RAEF (%)	42.1 ± 11.1	42.8 ± 9.8	0.91	50.3 ± 9.3	47.5 ± 10.8	0.01
Passive RAEF (%)	19.2 ± 5.8	11.4 ± 10.4	0.09	25.0 ± 15.1	16.8 ± 11.0	0.01
Active RAEF (%)	28.7 ± 11.0	35.2 ± 9.3	0.24	33.3 ± 6.4	37.0 ± 9.2	0.46

BSA—body surface area; CMR—cardiovascular magnetic resonance; LA—left atrium; LAEF—left atrial ejection fraction; LAV max—left atrial maximal volume; LAV min—left atrial minimal volume; LAV pac—left atrial pre-systolic volume; LVEDV—left ventricular end diastolic volume; LVEDVI—left ventricular end diastolic volume index; LVEF—left ventricular ejection fraction; LVESV—left ventricular end systolic volume; LVESVI—left ventricular end systolic volume index; LGE—late gadolinium enhancement; LVM—left ventricular myocardial mass; LVSV—left ventricular stroke volume; LVMI—left ventricular myocardial mass; LVSVI—left ventricular stroke volume index; RA—right atrium; RAEF—right atrial ejection fraction; RAV max—right atrial maximal volume; RAV min—right atrial minimal volume; RAV pac—right atrial pre-systolic volume; RVEDV—right ventricular end diastolic volume; RVEDVI—right ventricular end diastolic volume index; RVESV—right ventricular end systolic volume; RVESVI—right ventricular end systolic volume index; RVEF—right ventricular ejection fraction; RVSV—right ventricular stroke volume; RVSVI—right ventricular stroke volume index.

**Table 4:** CMR-FT derived strain parameters in patients with PPCM with and without LGE.

	PPCM without LGE n = 12 (67%)	PPCM with LGE n = 9 (43%)	*p*	DCM without LGE n = 12 (40%)	DCM with LGE n = 18 (60%)	*p*
Left ventricle						
LV GRS (%)	17.9 ± 5.8	9.2 ± 3.4	0.002	16.9 ± 2.7	11.9 ± 5.2	<0.01
LV GCS (%)	−13.3 ± 4.5	−8.0 ± 2.9	0.013	−11.9 ± 2.0	−8.5 ± 3.5	<0.01
LV GLS (%)	−12.3 ± 3.4	−6.6 ± 1.9	0.002	−9.1 ± 1.7	−6.8 ± 2.8	0.01
LV GRS rate (s^−1^)	1.1 ± 0.43	0.62 ± 0.28	0.015	0.86 ± 0.21	0.70 ± 0.21	0.04
LV GCS rate (s^−1^)	−0.78 ± 0.22	−0.46 ± 0.21	0.011	−0.62 ± 0.11	−0.47 ± 0.13	<0.01
LV GLS rate (s^−1^)	−0.61 ± 0.16	−0.47 ± 0.04	0.053	−0.42 ± 0.09	−0.46 ± 0.14	0.38
Right ventricle						
RV GRS (%)	17.9 ± 5.3	9.3 ± 3.6	0.002	17.1 ± 6.2	10.6 ± 10.0	0.03
RV GCS (%)	−11.1 ± 2.3	−6.5 ± 3.3	0.015	−11.7 ± 5.3	−6.1 ± 6.6	0.01
RV GLS (%)	−17.9 ± 7.3	−11.0 ± 5.5	0.06	−16.6 ± 6.7	−12.0 ± 7.0	0.09
RV GRS rate (s^−1^)	1.2 ± 0.52	0.74 ± 0.36	0.044	1.10 ± 0.48	0.64 ± 0.40	0.01
RV GCS rate (s^−1^)	−0.62 ± 0.3	−0.44 ± 0.23	0.21	−0.62 ± 0.19	−0.39 ± 0.27	0.01
RV GLS rate (s^−1^)	−1.09 ± 0.42	−0.92 ± 0.41	0.45	−1.01 ± 0.32	−0.78 ± −0.30	0.06
Left atrium						
LA GLS total (%)	22.7 ± 7.9	10.8 ± 6.6	0.006	26.3 ± 7.2	15.8 ± 8.4	<0.01
LA GLS passive (%)	11.1 ± 4.9	7.0 ± 4.8	0.11	12.0 ± 3.0	9.4 ± 4.7	0.12
LA GLS active (%)	12.5 ± 5.2	2.7 ± 2.3	<0.001	11.9 ± 2.1	6.8 ± 4.6	<0.01
LA SRS (s^−1^)	0.91 ± 0.33	0.50 ± 0.24	0.014	0.94 ± 0.45	0.64 ± 0.35	0.06
LA SRE (s^−1^)	−0.83 ± −0.43	−0.45 ± 0.30	0.06	−0.92 ± 0.63	−0.78 ± 0.45	0.50
LA SRA (s^−1^)	−0.72 ± 0.46	−0.66 ± 0.37	0.77	−0.74 ± 0.37	−0.56 ± 0.37	0.23
Right atrium						
RA GLS total (%)	27.1 ± 9.0	26.0 ± 11.4	0.83	32.0 ± 9.9	23.1 ± 7.5	0.01
RA GLS passive (%)	10.5 ± 5.3	12.2 ± 7.9	0.62	15.1 ± 6.6	9.1 ± 4.3	<0.01
RA GLS active (%)	16.7 ± 10.0	13.8 ± 5.5	0.51	11.9 ± 4.0	14.0 ± 7.0	0.37
RA SRS (s^−1^)	1.2 ± 0.41	1.1 ± 0.42	0.54	1.4 ± 0.42	0.99 ± 0.39	0.02
RA SRE (s^−1^)	−0.65 ± 0.29	−0.50 ± 0.37	0.39	−0.88 ± 0.69	−0.62 ± 0.32	0.25
RA SRA (s^−1^)	−0.79 ± 0.49	−1.2 ± 0.85	0.20	−0.79 ± 0.42	−0.96 ± 0.41	0.31

GCS—global circumferential strain; GLS—global longitudinal strain; GRS—global radial strain; LA—left atrium; LV—left ventricle; RA—right atrium; RV—right ventricle; SRA—late negative strain rate; SRE—early negative strain rate; SRS—peak positive strain rate.

## Data Availability

The data presented in this study are available upon request from the corresponding author.

## Supplementary Materials

The following are available online at https://www.mdpi.com/article/10.3390/diagnostics11101752/s1, Table S1: Intraobserver and interobserver reproducibility of feature tracking.
