# Supplementary material for: Cardiovascular Magnetic Resonance in Peripartum Cardiomyopathy: Comparison with Idiopathic Dilated Cardiomyopathy
Source: Diagnostics (Basel). 2021 Sep 24;11(10):1752. doi: 10.3390/diagnostics11101752 (PMC8535040; doi:10.3390/diagnostics11101752)
Supplement: Supplementary file 1 [file diagnostics-11-01752-s001.zip › diagnostics-1377342-supplementary.pdf]

Supplementary Table S1. Intra- and interobserver reproducibility of feature tracking.

	Interobserver variability			Intraobserver variability		
	Mean difference± SD	ICC (95%CI)	CoV (%)	Mean difference ±SD	ICC (95%CI)	CoV (%)
LV GRS (%)	1.08±1.98	0.95 (0.85-0.98)	7.1	0.46±0.99	0.98 (0.47-0.99)	8.9
LV GCS (%)	1.04±1.24	0.94 (0.70-0.98)	9.9	-0.68±0.62	0.99 (0.98-0.99)	-12.2
LV GLS (%)	0.67±2.39	0.85 (0.67-0.94)	13.4	0.77±1.20	0.92 (0.80-0.97)	-12.8
LV GRS rate (s^{-1})	-0.10±0.14	0.94 (0.85-0.97)	15.2	-0.27±0.15	0.98 (0.94-0.99)	13.7
LV GCS rate (s^{-1})	0.04±0.06	0.97 (0.93-0.99)	9.2	-0.14±0.06	0.94 (0.86-0.98)	-23.9
LV GLS rate (s^{-1})	-0.18±0.21	0.69 (0.22-0.88)	21.5	-0.18±0.10	0.87 (0.68-0.95)	-13.2
RV GRS (%)	0.58±4.55	0.81 (0.58-0.92)	23.4	2.70±2.71	0.93 (0.84-0.97)	17.0
RV GCS (%)	0.24±1.35	0.95 (0.87-0.98)	14.4	-1.08±0.67	0.99 (0.97-0.99)	-12.0
RV GLS (%)	1.45±5.58	0.78 (0.54-0.91)	19.5	-0.77±2.79	0.94 (0.85-0.97)	-17.18
RV GRS rate (s^{-1})	-0.48±0.44	0.73 (0.32-0.89)	43.1	-0.29±0.22	0.94 (0.85-0.98)	19.8
RV GCS rate (s^{-1})	0.16±0.34	0.50 (0.26-0.80)	25.2	1.18±0.67	0.92 (0.79-0.97)	-19.9
RV GLS rate (s^{-1})	0.69±3.18	0.95 (0.87-0.98)	-54.9	-0.03±0.42	0.88 (0.71-0.95)	-33.3
LA GLS total (%)	-3.43±4.15	0.92 (0.80-0.97)	30.7	5.21±3.76	0.93 (0.83-0.97)	34.4
LA GLS passive (%)	0.73±2.28	0.89 (0.75-0.96)	25.9	0.18±0.57	0.95 (0.86-0.98)	4.7
LA GLS active (%)	-0.53±1.52	0.98 (0.94-0.99)	16.5	0.61±1.34	0.96 (0.90-0.98)	16.3
LA SRS (s^{-1})	-0.05±0.15	0.83 (0.63-0.93)	21.6	-0.01±0.04	0.99 (0.98-0.99)	4.4
LA SRE (s^{-1})	0.07±0.23	0.91 (0.77-0.96)	-27.7	-0.13±0.28	0.84 (0.59-0.94)	-43.8
LA SRA (s^{-1})	-0.07±0.18	0.90 (0.75-0.96)	-20.7	-0.02±0.08	0.97 (0.92-0.99)	-8.7

RA GLS total (%)	4.04±4.79	0.90 (0.76-0.96)	17.9	2.00±2.33	0.96 (0.90-0.98)	8.4
RA GLS passive (%)	-0.46±2.78	0.94 (0.85-0.98)	16.6	0.22±1.90	0.94 (0.85-0.97)	11.6
RA GLS active (%)	-6.83±4.83	0.69 (0.22-0.88)	16.2	3.45±4.84	0.73 (0.31-0.89)	31.2
RA SRS (s^{-1})	-0.05±0.25	0.82 (0.55-0.93)	15.9	0.05±0.25	0.87 (0.68-0.95)	16.9
RA SRE (s^{-1})	0.18±0.33	0.69 (0.22-0.88)	-36.8	-0.14±0.23	0.92 (0.79-0.97)	-31.1
RA SRA (s^{-1})	-0.06±0.20	0.83 (0.58-0.93)	-18.5	-0.01±0.20	0.87 (0.68-0.95)	-17.4

CoV- coefficient of variance, GCS – global circumferential strain, GLS –global longitudinal strain, GRS - global radial strain, ICC – interclass correlation coefficient, LA – left atrium, LV – left ventricle, RA – right atrium, RV – right ventricle, SD – standard deviation, SRA - late negative strain rate, SRE - early negative strain rate, SRS - peak positive strain rate.
